# ROS-independent Nrf2 activation in prostate cancer

**DOI:** 10.18632/oncotarget.18724

**Published:** 2017-06-28

**Authors:** Ilaria Bellezza, Paolo Scarpelli, Salvatore V. Pizzo, Silvia Grottelli, Egidia Costanzi, Alba Minelli

**Affiliations:** ^1^ Department of Experimental Medicine, University of Perugia, Perugia, Italy; ^2^ Duke University School of Medicine, Durham, NC, USA

**Keywords:** GRP78/BiP, Nrf2, Akt, Anti-GRP78/BiP antibody, dexamethasone

## Abstract

In prostate cancer, oxidative stress and the subsequent Nrf2 activation promote the survival of cancer cells and acquired chemoresistance. Nrf2 links prostate cancer to endoplasmic reticulum stress, an event that triggers the unfolded protein response, aiming to restore cellular homeostasis as well as an adaptive survival mechanism. Glucose-regulated protein of 78 kD /immunoglobulin heavy chain binding protein (GRP78/BiP) is a key molecular chaperone in the endoplasmic reticulum that, when expressed at the cell surface, acts as a receptor for several signaling pathways enhancing antiapoptotic and proliferative signals. We showed GRP78/BiP translocation to PC3 cell surface in the presence of tunicamycin, an ER stress inductor, and demonstrated the existence of a GRP78/BiP-dependent non-canonical Nrf2 activation, responsible for increased resistance to ER-stress induced apoptosis. We found that, even in the absence of ROS production, tunicamycin causes Nrf2 activation, and activates Akt signaling, events bulnted by anti-GRP78/BiP antibody treatment. The presence of GRP78/BiP at the cell surface might be exploited for the immunotherapeutic strategy of prostate cancer since its blockage by anti-GRP78/BiP antibodies might promote cancer death by suppressing some of the several molecular protective mechanisms found in aggressive cancer cells.

## BACKGROUND

Oxidants and oxidative stress–inducing agents activate the transcription factor NF-E2-related factor 2 (Nrf2) which controls the fate of the cell by up-regulating the transcription of stress-response genes thus enhancing the antioxidant cell defense and maintaining cellular redox homeostasis [1]. Elevated levels of reactive oxygen species (ROS) in normal cells are harmful and can lead to the induction of cell death by necrosis and/or apoptosis. However, acute and short exposure to high levels of ROS or chronic exposure to low levels of ROS can increase cell proliferation and accelerate tumorigenesis by altering the expression of growth factors and proto-oncogenes [2–4]. In prostate cancer (PCa), oxidative stress is one of the several hallmarks of the aggressive phenotype since oxidative stress is associated with PCa development, progression and the response to therapy [5]. Therefore, activation of Nrf2 pathway, leading to an adaptive response, was firstly proposed as a promising strategy for cancer prevention [6]. However, the negative results of several antioxidant-supplemented clinical trials [7], questioned the relationship between oxidative stress and the activation of survival pathways in malignant prostate. Furthermore, Nrf2 protects not only normal cells from transforming into cancer cells, but also promotes the survival of cancer cells [8]. Moreover, Nrf2 and its downstream genes are over-expressed in many cancer cell lines and human cancer tissues, as well as being up-regulated in resistant cancer cells and responsible for acquired chemoresistance [9–10]. Nrf2 and its activation, besides driving the effects of cellular oxidants and toxic compounds in the cells, as a direct substrate of the PERK branch of the unfolded protein response [11] definitely links PCa to endoplasmic reticulum (ER) stress [12–13].

Prostate cancer is one of the most common cancer in male population, representing 20 % of the newly diagnosed malignancies in Italy in 2015 [14]. Androgen ablation is the standard therapeutic option which leads to an initial regression followed, in the vast majority of the cases, by a tumor relapse into castration resistant PCa (CRPC), a particularly aggressive phenotype for which there is currently no therapeutic treatment available [15]. PCa, as a solid tumor, deals with events that, by interfering with the ER, lead to ER stress [15]. To restore ER homeostasis, the cells activate several signaling pathways, known as the unfolded protein response (UPR). UPR activation represents an adaptive survival mechanism [16]. In PCa, UPR marker gene activation and tumor progression are linked either by a negative correlation in model systems *in vitro* [12], or by a positive association that involves androgens and androgen receptor (AR) [17]. Among the several players of UPR, glucose-regulated protein of 78 kD /immunoglobulin heavy chain binding protein (GRP78/BiP) is a key molecular chaperone in the ER, where it presides the folding and assembly of newly synthesized proteins. Elevated levels of GRP78/BiP characterize several cancer cell lines and human cancers with a close association with metastases and resistance to chemotherapy [18]. Indeed, under ER stress conditions, it can be expressed at the cell surface, acting as a receptor for several signaling pathways that control/enhance antiapoptotic and proliferative signals [19–20]. AR negative PC3 cells, treated with tunicamycin (TM), up-regulated GRP78/BiP mRNA levels [21], and, when exposed to thapsigargin, relocalised GRP78/BiP on the membrane [22].

In the present study, we showed GRP78/BiP translocation to the cell surface in the presence of TM, an ER stress inductor. We aim to investigate whether GRP78/BiP translocation is responsible for PC3 resistance to cell death via a non-canonical Nrf2 activation.

## RESULTS

### Tunicamycin causes Nrf2 activation in the absence of increased levels of ROS

**Figure 1:**
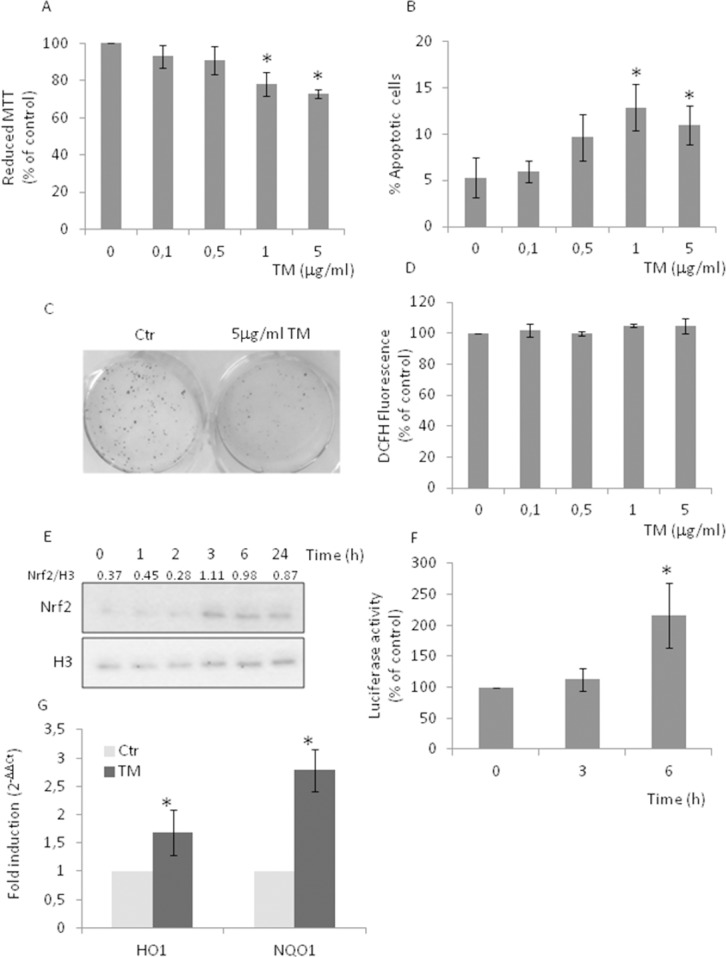
Tunicamycin causes Nrf2 activation in the absence of increased levels of ROS PC3 cells were treated with increasing concentrations (0-5μg/ml) of TM for 24 h. **(A)** Cell viability as detected by MTT assay; **(B)** percentage of apoptotic cells as detected by PI staining and FACS analysis; **(C)** clonogenic assay, in the presence of 5μg/ml TM; **(D)** ROS levels as detected by DCFH fluorescence; **(E)** Nrf2 nuclear levels as detected by western blotting, histone H3 was used as loading control. **(F)** Nrf2 activation as detected by luciferase assay. Control values (mean ± S.D., n = 6) are given as 100%. **(G)** HO-1 and NQO-1 expression as determined by qPCR. Expression was normalised to GAPDH and reported as 2^− ΔΔCt^. Relative mRNA level of untreated cells was assumed to be 1. *p < 0.05 vs. control cells.

Protein folding occurring in the ER drives the production of reactive oxygen species (ROS), which, in turn, can cause ER stress and trigger the UPR, one of the several pathogenetic mechanisms of prostate cancer initiation and progression [23–24]. To investigate the molecular mechanism underlying the aggressive disease phenotype, we used the AR negative PC3 cell line and studied their response to the treatment with increasing concentrations of tunicamycin (TM) (Figure 1). We observed a moderate decrease in cell viability (Figure 1A) as well as a moderate increase in the number of apoptotic cells (Figure 1B), both suggestive of a mild toxicity of the ER stressor. The clonogenic assay confirmed the presence of viable PC3 cells after treatment with 5 μg/ml TM (Figure 1C). Given that oxidative stress is one of the hallmarks of the aggressive phenotype [2–4], and the existence of a cross talk between UPR and Nrf2 [11], we then analyzed the effects of TM treatment on ROS production (Figure 1D), nuclear translocation (Figure 1E) and transcriptional activity of Nrf2 (Figure 1F), and transcription of Nrf2-master genes (Figure 1G). TM did not increase ROS production while causing a robust Nrf2 activation and the up-regulation (approx. 2 folds) of Nrf2-driven genes Hemeoxygenase-1 (HO-1) and NADPH-quinone oxidoreductase-1 (NQO1). High levels of basal nuclear Nrf2 were observed in PC3, as compared with MDAPCa2b, an androgen sensitive cell line (Supplementary Figure 1A). Results support the activation of the redox-sensitive transcription factor Nrf2 as one of several culprits of cancer cell death.

### Tunicamycin induces the activation of the IRE1α arm

**Figure 2:**
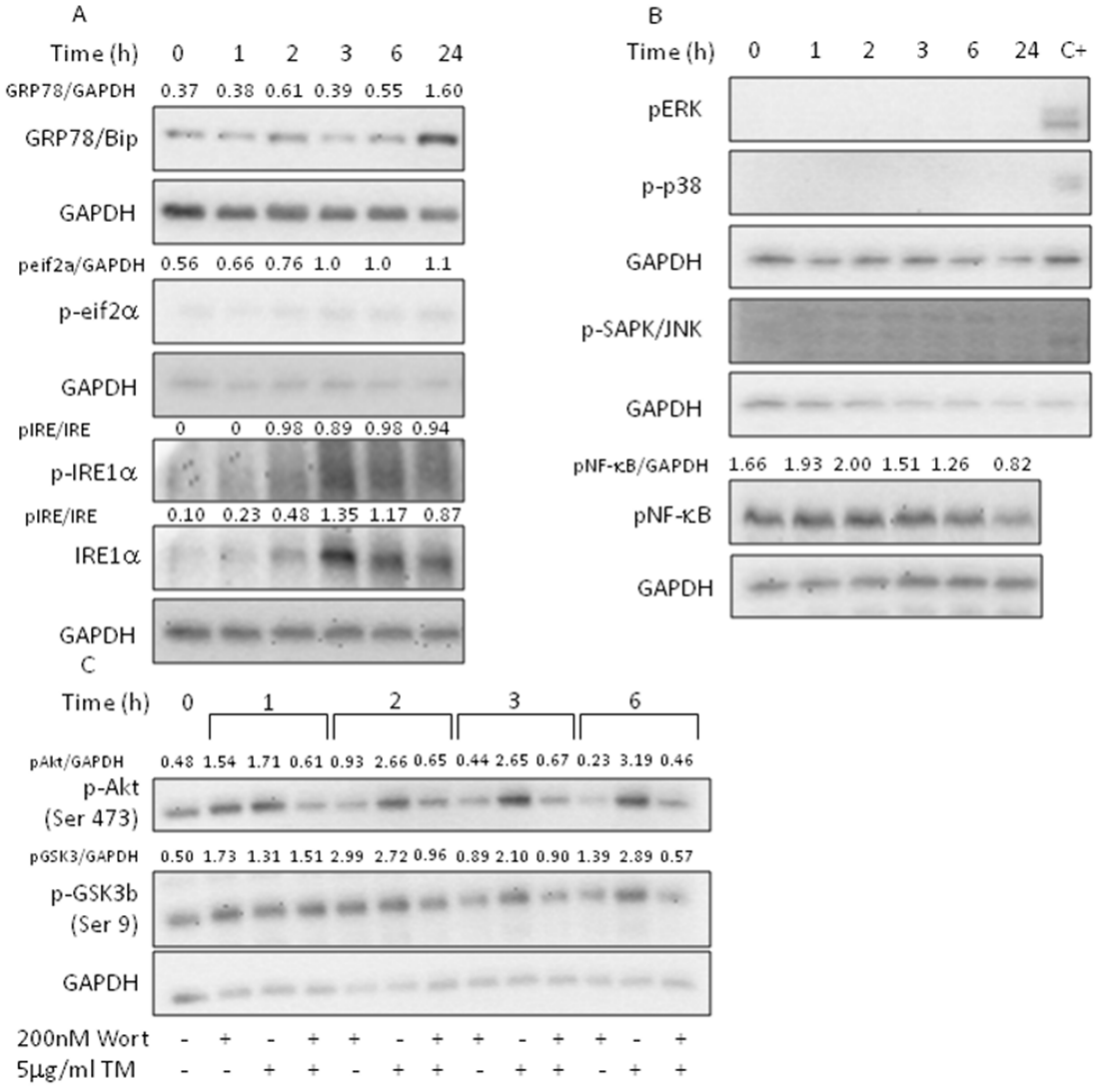
Tunicamycin activates AKT signaling PC3 cells were treated with 5μg/ml TM for the indicated time, collected and total extract subjected to western blotting with the indicated antibodies. Wortmannin was added to cell culture 1h prior to TM treatment. **(A)** Analysis of UPR markers; **(B)** analysis of MAPK activation; **(C)** analysis of Akt pathway activation. GAPDH was used as loading control. One out of four independent experiments giving similar results is shown.

The highly integrated and regulated UPR signal transduction pathways are triggered by three proteins residing in the ER membrane: inositol requiring-enzyme 1 alpha (IRE1α), activating transcription factor 6 alpha (ATF6α) and protein kinase RNA-like ER kinase (PERK). These ER sensors, under normal and physiological conditions, are kept in an inactive state by GRP78/BiP, which, upon several ER stressing stimuli, dissociates from the sensors and activates the UPR signaling pathways [16]. PC3 cells treated with TM showed a late increase in GRP78/BiP levels, probably indicative of the activation of the ATF6α branch, absence of PERK-mediated phosphorylation of eif2α at each considered time, whereas phosphorylation of IRE1α was detected at 2-3h and remained sustained up to 24h (Figure 2A). Results indicate the activation of the adaptive IRE1α and ATF6α branches of the UPR while simultaneously inhibiting the PERK pathway.

### Tunicamycin-induced IRE1α activation fails to activate MAPK cascades

Phosphorylated IRE1α acts as a stress-specific scaffold on the cytosolic side of ER and associates with TRF-receptor associated factor 2 (TRAF2) to activate several kinases, which, in turn, contribute to cell fate decision during ER stress by acting on Nrf2 and NF-κB [23]. The treatment of PC3 cells with TM failed to activate extracellular signal-regulated kinase (ERK), c-Jun N-terminal kinase (JNK), p38 MAPK, and NF-κB (Figure 2B), indicating that the activation of all three MAPK cascades were not responsible for Nrf2 modulation.

### Tunicamycin activates Akt signaling

The phosphatidylinositol 3’-kinase (PI3K)/Akt signaling pathway regulates cell survival during oxidative stress by activating Nrf2 [25–26]. Given that Akt is overexpressed in prostate cancers [27] and that activated Akt promotes cell survival [28–29], to better understand the mechanism underpinning Nrf2 activation, we investigated the effects of TM treatment on Akt signaling pathway (Figure 2C). We found that TM increased the levels of phosphorylated Akt with a time-course indicating a maximal activation at 3h lasting up to 6h. Concomitantly, we observed GSK3β phosphorylation, thus inhibiting the apoptosis-inducing kinase activity. Pretreatment of the cells with wortmannin, a PI3-kinase inhibitor, showed that activation of Akt and inactivation of GSK3β are PI3K dependent. These results indicate that Nrf2 activation, under our experimental condition, can, at least partly, be attributed to the activation of Akt.

### Tunicamycin treatment induces the translocation of GRP78/Bip to the cell surface

**Figure 3:**
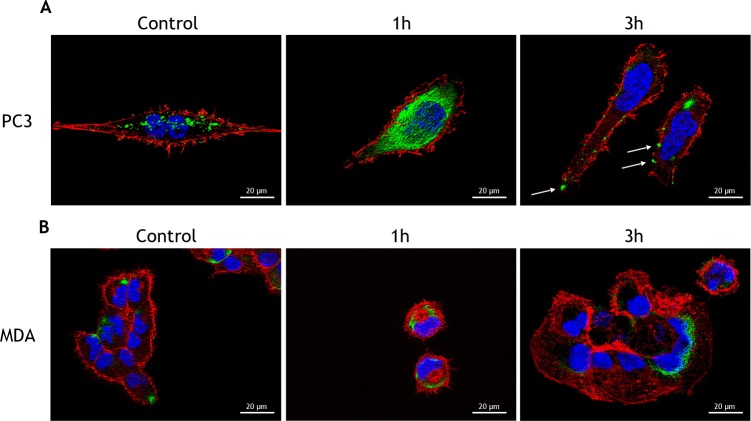
Tunicamycin treatment induces the translocation of Bip to the cell surface **(A)** PC3 cells and **(B)** MDAPCa2b cells were treated with 5μg/ml TM for the indicated times and used for detection of GRP78/BiP (green) by immunofluorescence. Cytoskeletal actin was visualized by AlexaFluor555-conjugated phalloidin staining (red) and nuclei were counterstained with DAPI (blue). Magnification: 63×.

**Figure 4:**
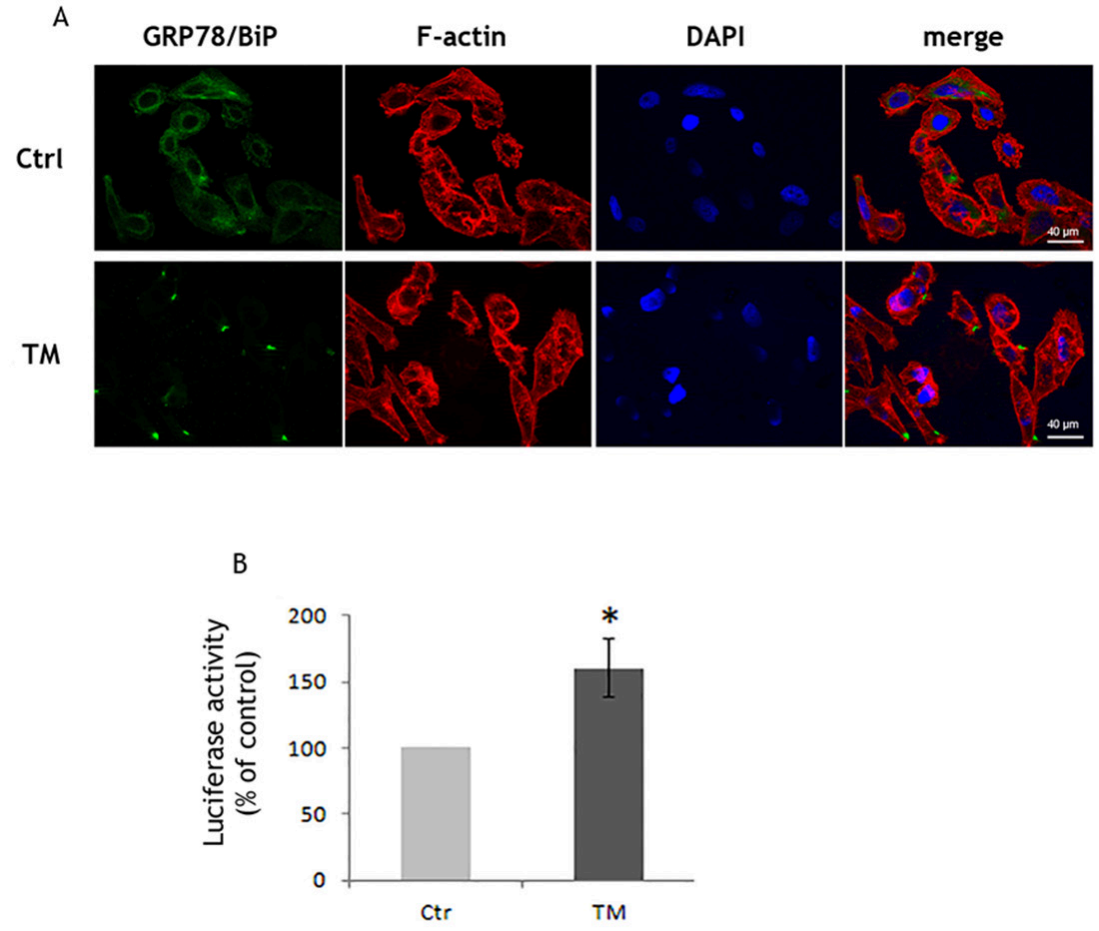
Low tunicamycin concentration induces the translocation of Bip to the cell surface PC3 cells were treated with 1μg/ml TM and **(A)** after 3 h used for the detection of GRP78/BiP (green) by immunofluorescence. Cytoskeletal actin was visualized by AlexaFluor555-conjugated phalloidin staining (red) and nuclei were counterstained with DAPI (blue). Magnification: 40×; **(B)** after 6h used for the determination of Nrf2 activation by luciferase assay. Control values (mean ± S.D., n = 6) are given as 100%.

GRP78/BiP is a resident ER chaperone which appears at the surface of many cancer cells, where it is involved in the activation of pro-proliferative/anti-apoptotic signaling mechanisms [30–33]. Although it is known that PC3 cells do not express GRP78/BiP on their cell surface, thapsigargin treatment was shown to induce GRP78/BiP membrane localization [22]. Therefore, we anticipated that the treatment of PC3 with TM could also relocalizeGRP78/BiP on the cell surface. We found that a 3h treatment with TM caused GRP78/BiP translocation to the cell membrane, thus suggesting a mechanism for PC3 refractoriness to TM (Figure 3A). To support the hypothesis that translocation of GRP78/BiP to the cell surface is responsible for increased survival of the cells and drug resistance, we used AR positive MDAPCa2b cells that, when treated with TM, responded with decreased cell survival, increased apoptosis, and increased ROS production (Supplementary Figure 1B-1E). Indeed, in MDAPCa2b treated with TM, we did not observe GRP78/BiP translocation to the membrane (Figure 3B). The treatment of PC3 cells with 1μg/ml TM caused GRP78/BiP translocation at the cell surface (Figure 4A) and Nrf2 activation (Figure 4B), thus confirming that the observed effects were not due to tunicamycin toxicity. The secretory pathway, representing the first step in the protein export from the ER, is mediated by coatomer protein II (COPII)-coated vesicles at ER exit sites. The conserved large hydrophilic protein Sec16 localizes at the ER exit sites and plays a pivotal role in modulating the COPII dynamics [34]. In PC3 and MDAPCa2b cells treated with TM, we observed the up-regulation of Sec16L mRNA levels, thus confirming the activation of the secretory pathway by TM (Supplementary Figure 1F).

### Anti-GRP78/BiP C-terminal domain antibody treatment blunts Nrf2 activation

**Figure 5:**
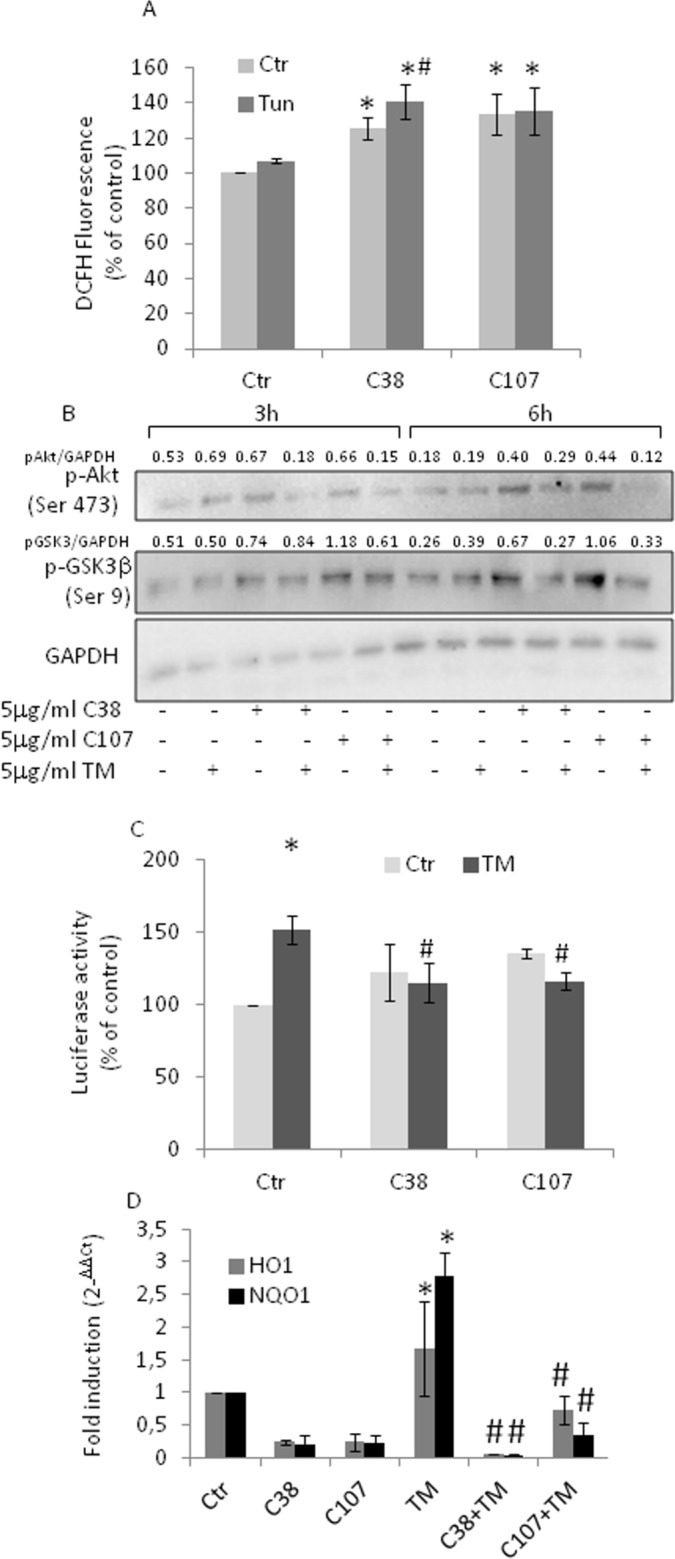
Anti- GRP78/BiP C-terminal domain antibody treatment blunts Nrf2 activation PC3 cells were treated with 5μg/ml of TM 10 min prior to antibody addition (5μg/ml C38 or C107) and grown for the indicated times. **(A)** ROS levels as detected by DCFH fluorescence. **(B)** Total cell extract subjected to western blotting with the indicated antibodies. GAPDH was used as loading control. One out of four independent experiments giving similar results is shown. **(C)** Nrf2 activation as detected by luciferase assay; Control values (mean ± S.D., n = 4) are given as 100%. **(D)** HO-1 and NQO-1 expression as determined by qPCR. Expression was normalised to GAPDH and reported as 2^− ΔΔCt^. Relative mRNA level of untreated cells was assumed to be 1. *p < 0.05 vs. control cells; # p<0.05 vs. TM-treated cells.

The activation of antioxidant and anti-apoptotic signaling is a protective response since it establishes a shield over death signals in cancer cells. The translocation of GRP78/Bip to the cell surface of PCa leads to cell survival and proliferation, thus contributing to masking several stresses [28–29, 35]. To determine whether Nrf2 activation and the subsequent redox adaptation, a strategy frequently used by cancer cells to survive and become resistant to several anticancer agents, might be related to the response of PC3 cells to TM treatment, we exposed PC3 cells to GRP78/BiP-antibody and investigated the effects of TM. Under these experimental conditions, we found an increase in ROS levels (Figure 5A), changes in the phosphorylation of Akt and GSK3β, indicative of a reduced activation of Akt (Figure 5B). Moreover, the Nrf2 transcriptional activity reverted to control values (Figure 5C) as well as Nrf2-driven gene expression (Figure 5D). Results indicate that Nrf2 activation can be, at least partially, due to GRP78/BiP translocation.

### Dexamethasone treatment of PC3 cells do not affect GRP78/BiP translocation

**Figure 6:**
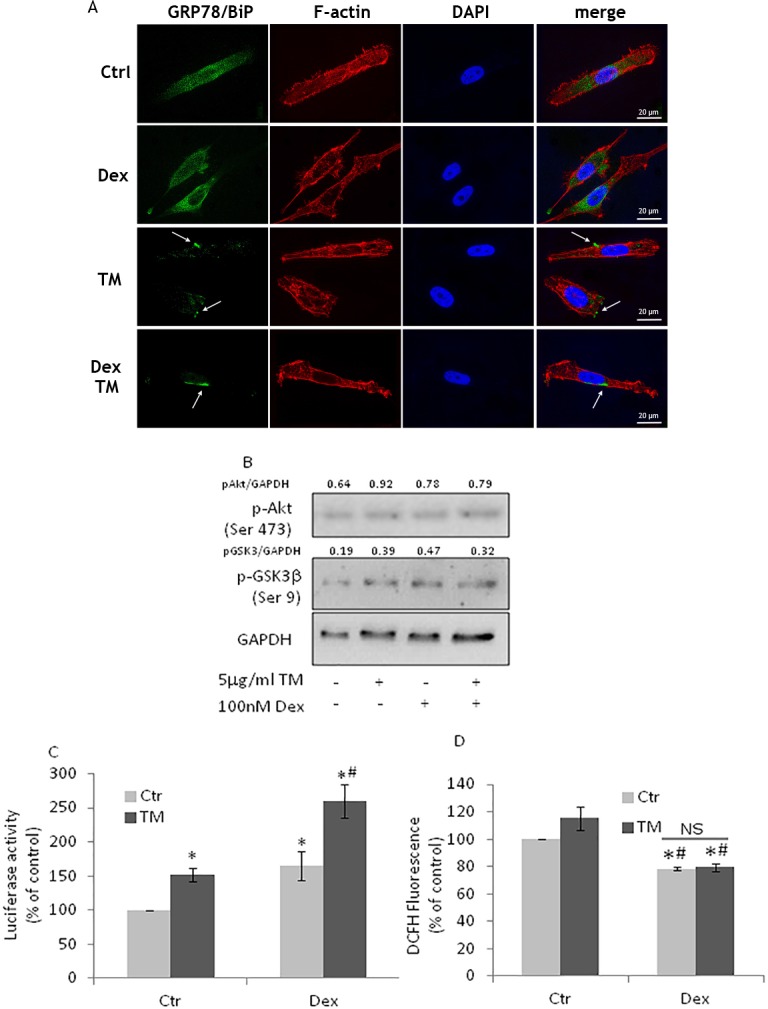
Dexamethasone treatment of PC3 cells does not affect GRP78/BiP translocation **(A)** PC3 cells, treated with100nM Dexamethasone (Dex) for 72h, were exposed to 5μg/ml TM for 3h and used for detection of GRP78/BiP (green) by immunofluorescence. Cytoskeletal actin was visualized by AlexaFluor555-conjugated phalloidin staining (red) and nuclei were counterstained with DAPI (blue). Magnification: 63×. PC3 cells, after Dex treatment, were exposed to TM for 6h and used for **(B)** western blotting analyses with the indicated antibodies. GAPDH was used as loading control. One out of four independent experiments giving similar results is shown; **(C)** Nrf2 activation as detected by luciferase assay; **(D)** PC3 cells, after Dex treatment, were exposed to TM for 24h and ROS levels were detected by DCFH fluorescence; control values (mean ± S.D., n = 4) are given as 100%. *p < 0.05 vs. control cells; # p<0.05 vs. TM-treated cells.

The combination of chemotherapy and dexamethasone is a classic treatment for CRPC patients [36] thus we investigated the effects of dexamethasone treatment on TM-treated PC3 cells. We found that PC3 cells still exposed GRP78/BiP on their surface (Figure 5A) and that Akt pathway was still active (Figure 5B) as well as Nrf2 (Figure 6C) resulting in unchanged ROS levels (Figure 6D). The results indicate that dexamethasone treatment does not modify Nrf2 adaptive responses.

## DISCUSSION

Here we have shown in PC3 cells, an AR negative PCa cell line, that translocation of GRP78/BiP to the cell surface enhances Nrf2 activation thus contributing to cells survival. Cancer is a complex biological process characterized by several hallmarks, i.e. biological capabilities that increase survival by evading cell death signaling and immune destruction in a new tissue microenvironment [37]. PCa is the second-leading cause of cancer-related mortality in men in Western countries, representing 20 % of the newly diagnosed malignancies in Italy in 2015 [14]. PCa initially responds to surgical or chemical depletion of the AR ligand. However, castration induces several adaptive responses which, combined with genomic and epigenomic mutations [38–39], lead to the progression, in approx. 18 months, to a more aggressive phenotype, i.e. CRPC. These cancer cells are provided with a high-grade plasticity, thus a single adaptive pathway is not the only responsible for the apoptosis inhibition and treatment resistance [40]. Among the various adaptive/survival responses, the transcription factor Nrf2 and its network plays a crucial role, going from antioxidant genes to metabolic and proteostasis genes, and autoregulatory pathways [41]. Because of this extended coverage, Nrf2 employs more than one regulatory strategy for transcriptional activation in response to stress. Nrf2 activation is tightly bound to increase on ROS level. Indeed, in normal cell homeostasis, Nrf2 is constantly ubiquitinated by the E3 ligase Keap-1 and degraded. Upon oxidative or electrophilic stress, Nrf2, detached from Keap-1, is translocated to the nucleus, where, by binding to the antioxidant/electrophile response element (ARE/EpRE) in target gene promoters, up-regulates the expression of downstream Nrf2 target genes that coordinate the adaptive responses [1, 42]. However, Nrf2 activation can be harmful in cancer situations [3, 8, 43]. Mutations that disrupt the Nrf2-Keap-1 interaction, cause a constitutive activation of this transcription factor, and such mutations are observed in lung, colorectal, and prostate cancers [44–46]. Undeniably, the sustained activation of Nrf2 produces a supportive environment for survival and therapeutic resistance. AR negative PC3 cells, used in this study, are characterized by high basal nuclear levels of Nrf2, a condition that could explain the lack of ROS production as well as the low apoptotic response that we observed after treating PC3 cells with an ER stressor, TM. Nevertheless, under ER stress conditions, we observed an increased transcriptional activity of Nrf2, with a concomitant increase in HO1 and NQO1 gene expression, which could not be attributed to the PERK branch of the UPR pathway. Therefore, we suggest the existence of a non canonical signaling mechanism that may increase Nrf2 activation in the absence of increased levels of ROS under an ER stress setting. As a solid tumor, PCa often deals with microenvironmental factors that, by compromising the ER function, lead to ER stress. In turn, the stress elicits UPR and all the related cytoprotective mechanisms aimed to favoring tumor growth [23–24]. UPR activation as an adaptive survival mechanism is found in gastric cancer, in malignant glioma, and in chronic lymphocytic leukemia [47–49]. Although at times the relationship between UPR markers and adaptive survival has been debated [12], several studies have shown a positive link between UPR markers and androgen-driven PCa [13, 17, 24]. Moreover, androgens activate the IRE1α-XBP1 arm of UPR and concurrently inhibit the PERK-eIF2α pathway [17]. In AR negative PC3 cells, we found that TM did not activate the PERK-eIF2α signaling whereas it increased the protein level of GRP78/BiP, a target gene of the ATF6α arm, and activated the IRE1α arm. Although the role of the IRE1α pathway in AR-negative cell lines still needs to be further investigated, it is now accepted that the IRE1α arm plays a pro-survival role in PCa [17, 24]. The expression of molecular chaperones provides numerous types of cancer with pro-survival aptitude [50]. In PCa, GRP78/BiP is one of the most studied molecular chaperone and the increases in its expression are correlated with PCa recurrence and short survival [51–52]. In several cancers, as well as in PCa, GRP78/BiP translocates to cell surface thus constituting a cancer-specific cell surface marker, tightly related to hormonal resistance of PCa [28, 33]. The PI3K/Akt pathway is significantly involved in multiple biological responses that promote survival thus the increased phosphorylation of Akt in prostate cancer tissues frequently prevents apoptosis and contributes to tumor progression [53]. GRP78/BiP, when expressed at the cell surface of tumor cells, enhances the Akt signaling thus contributing to cell survival and aggressive phenotype. Indeed, the highly metastatic 1-LN cells, derived from less metastatic PC3 cells, express GRP78/BiP on their cell surface [22, 29, 35]. By treating PC3 cell with TM, we therefore obtained cells with a distinctive marker of high metastatic potential. Given previous studies, activation of surface–bound GRP78/BiP will trigger the activation of Akt, which by inactivating GSK3β, leads to the reinforcement of Nrf2-mediated defense mechanism. Our study, by using wortmannin, a PI3K inhibitor, confirms that the role of Akt in PC3 is linked to the activation of the Nrf2/ARE transcriptional regulation in the presence of translocated GRP78/BiP. Indeed, when we specifically targeted cell surface GRP78/BiP with C-terminal directed antibodies, we observed a decreased Nrf2 activation, concomitant to a decreased activation of Akt and a decreased inactivation of GSK3β. The active GSK3β enhances Nrf2 degradation thus weakening PC3 cellular defense. Our proposal, i.e. that translocated GRP78/BiP could enhance Nrf2 activation, was substantiated by using MDAPCa2b cell line, characterized by the lack of GRP78/BiP translocation upon TM treatment. These cells responded to the TM treatment with a decreased cell survival, an increased apoptosis, and an increased ROS production due to the lack of Nrf2 activation. Finally, we observed that dexamethasone treatment, commonly used to treat CRPC patients [36], did not abrogate the translocation of GRP78/BiP to the cell surface and every other described cellular response to TM.

In conclusion, the preferential expression of GRP78/BiP on the surface of cancer cells might be exploited for immunotherapeutic approaches of prostate cancer. It is known that antibodies to the carboxyl terminal domain of GRP78 promote cancer cell death [22] as also observed in the present study. Indeed, by decreasing Nrf2 activation, this strategy could remove some of the several molecular protective mechanisms found in aggressive cancer cells.

## MATERIALS AND METHODS

### Materials

All the reagents, unless otherwise stated, were from Sigma–Aldrich (St. Louis, MO). All the antibodies, unless otherwise stated were from Santa-Cruz Biotechnology (Santa Cruz, CA). Antibodies C38 and C107 were validated as described [20].

### Cell culture and treatment

AR negative PC3 and AR positive MDAPCa2b cell lines were obtained from American Type Tissue Culture Collection (Rockville, MD). Cells were grown in RPMI 1640 medium (Lonza, Milano, Italy) supplemented with 10% foetal bovine serum (FBS), glutamine (2 mM) and antibiotics (penicillin 100 U/ml and streptomycin 100 μg/ml). Sub-confluent cells were exposed to increasing concentrations of tunicamycin (0.1-5 μg/ml) for the indicated time. Wortmannin was added to cell culture 1h prior to tunicamicyn, anti GRP78/Bip antibodies (C38 and C107) were added 10min after tunicamycin treatment. In some experiments, cells were grown for 72h in the presence of dexamethasone and then treated with tunicamycin for the indicated times.

### MTT viability assay

Cell viability was measured using the MTT assay. 0.5 mg/ml MTT (3-(4,5-dimethylthiazol-2-yl)-2,5-diphenyltetrazolium bromide) was added to each well for 4 h, lysis buffer (10% SDS, 0,01 M HCl) was then added and cells lysed at 37°C overnight. Samples were measured with an automatic microplate reader (Seac, Firenze, Italy) at 550nm. Experiments were performed at least three times with six samples in each experimental group. Results were expressed as percentages (%) of reduced MTT, assuming the absorbance of control cells as 100% [54].

### Apoptosis determination by flow cytometry

Apoptosis was detected by propidium iodide (PI) (50 μg/ml in 0.1% sodium citrate plus 0.1% triton X-100) addition. The PI fluorescence of individual nuclei was measured by flow cytometry using standard FACScan equipment (Becton Dickinson, Franklin Lakes, NJ). The data were recorded in a Hewlett Packard (H9 9000, model 310, Palo Alto, CA) computer. The percentage of apoptotic cell nuclei (sub-diploid DNA peak in the DNA fluorescence histogram) was calculated with specific FACScan research software (Lysis II). At least 10,000 events were analysed in each sample [55].

### Measurement of intracellular fluorescence

The DCFH-DA method was used to detect the levels of intra-cellular reactive oxygen species (ROS). Cells were treated as described and DCFH-DA (30μM) was added into the medium for a further 30 min at 37°C. The fluorescence of 2’,7’-dichlorofluorescein was detected at 485 nm excitation and at 535 nm emission, using a TitertekFluoroscan II (Flow Laboratories, McLean, USA). Results were expressed as % of the control DCF fluorescence [54].

### Western blotting

Cells were lysed in boiling Laemmli sample buffer or processed with NE-PER(R) Nuclear and Cytoplasmic Extraction Reagents (Pierce Biotechnology, Rockford, IL) according to manufacturer’s instruction. Protein samples were electrophoresed on SDS polyacrylamide gels and transferred to nitrocellulose membranes at 100 V for 1 hr. Membranes were probed with the indicated antibodies (Supplementary Table 1), which were detected using HRP-based chemiluminescence (ECL, Pierce Biotechnology, Rockford, IL) [56]. Densitometric analises were performed with Image J software.

### Clonogenic assay

Cells were seeded at a density of 500 cells/dishes in 9.5 cm^2^ well. After a 7 days incubation at 37°C, the colonies were fixed with 3.7% paraformaldehyde, stained with crystal violet (0.5% w/v) and counted using a stereomicroscope.

### Immunofluorescence

Cells, seeded on glass coverslips, were fixed with 4% paraformaldehyde in PBS (Chem Cruz) for 20 min at room temperature. After blocking non-specific binding sites with 3% BSA in PBS for 1h, cells were incubated overnight at 4°C with anti GRP78 antibody (C38) (1:100) and then with anti mouse Alexafluor 488 secondary antibody (1:500). After washings, F-actin was stained with Alexafluor 555-Labelled phalloidin (1:200) for 30 minutes at room temperature. Cells were washed with PBS, cell nuclei were counter-stained with 4,6-diamidino-2-phenylindole (DAPI) and slides were mounted using glass coverslips and Prolong Diamond mounting medium (Life Technologies) for permanent sealing. Images were captured using a Zeiss Axio Observer. Z1 inverted microscope, equipped with Apotome filter and Axiocam MRm camera detection system Zeiss using a 63x/1.25 oil Plan/neofluar objective and a 40x/0.75 plan/neofluar objective. To improve axial resolution and focal planes separation, Apotome filter was enabled, set to “strong” with noise reduction set to “Off”. All the images, were processed for visual improvement, with the application of a 5,6 pixel 160 point sharpening mask and histograms were additionally stretched to increase contrast (Ps CC - Adobe). No in-silico extra noise reduction treatment was applied to the images.

### RNA isolation, reverse transcription and RT- PCR

Total RNA was isolated with TRI Reagent according to the manufacturer’s instructions and cDNA was synthesised using PrimeScript RT reagent Kit (Takara, Mountain View, CA). Real time PCR was performed using the QuantStudio 3 detection system (Applied Biosystem, Foster City, CA) and SYBR Green chemistry. Primers are listed in Supplementary Table 2. SYBR Green RT-PCR amplifications were carried out in a 96-well plate in a 25 μl reaction volume that contained 12.5 ml of SYBR® Green JumpStart™ Taq ReadyMix, 400 nM forward and reverse primers, and 5 to 40 ng of cDNA. In each assay, no-template controls were included and each sample was run in triplicates. Mean of Ct values of the samples was compared to the untreated control sample and GAPDH used as internal control. The n-fold differential ratio was expressed as 2^−ΔΔCt^.

### Transfection and luciferase reporter assays

Cignal ARE Dual-luciferase reporter kit (CCS-5020, Qiagen, Milan, IT) was used to transfect PC3 and MDA cells following manufacturer’s instructions. Transient transfections were carried out using Attractene transfection reagent (Qiagen, Milan, IT) according to the manufacturer’s instructions. Cell extracts were prepared 24 h after transfection and Luciferase activities in lysates were measured using Luminoskan (Thermo Electron Corporation, Waltham, MA) luminometer. Quantification of Firefly and Renilla luciferase activities was performed with the Dual Luciferase Reporter Assay System (Promega, Madison, WI). The relative firefly luciferase activity was calculated by normalizing transfection efficiency. The luciferase activity was expressed as arbitrary unit of light intensity.

### Statistical analysis

All studies were performed at least in triplicate, separate experiments and expressed as mean ± S.D. Data were analysed for statistical significance by Student’s t-test. When appropriate data were analysed by two-way ANOVA test with Sidak’s correction for multiple comparison. *P-values <0.05 were considered significant.

## SUPPLEMENTARY MATERIALS FIGURE AND TABLES



## Abbreviations

AR: androgen receptor
ARE/EpRE: antioxidant/electrophile response element
ATF6α: activating transcription factor 6 alpha
COPII: coatomer protein II
CRPC: castration resistant PCa
ER: endoplasmic reticulum
ERK: extracellular signal-regulated kinase
GRP78/BiP: glucose-regulated protein of 78 kD /immunoglobulin heavy chain binding protein
HO-1: Hemeoxygenase-1
IRE1α: inositol requiring-enzyme 1 alpha
JNK: c-Jun N-terminal kinase
NQO1: NADPH-quinone oxidoreductase-1
Nrf2: NF-E2-related factor 2
PCa: prostate cancer
PERK: protein kinase RNA-like ER kinase
PI3K: phosphatidylinositol 3’-kinase
ROS: reactive oxygen species
TM: tunicamycin
TRAF2: TRF-receptor associated factor 2
UPR: unfolded protein response
