# Supplementary material for: ROS-independent Nrf2 activation in prostate cancer
Source: Oncotarget. 2017 Jun 28;8(40):67506–18. doi: 10.18632/oncotarget.18724 (PMC5620189; doi:10.18632/oncotarget.18724)
Supplement: Supplementary file 1 [file oncotarget-08-67506-s001.pdf]

ROS-independent Nrf2 activation in prostate cancer

SUPPLEMENTARY MATERIALS

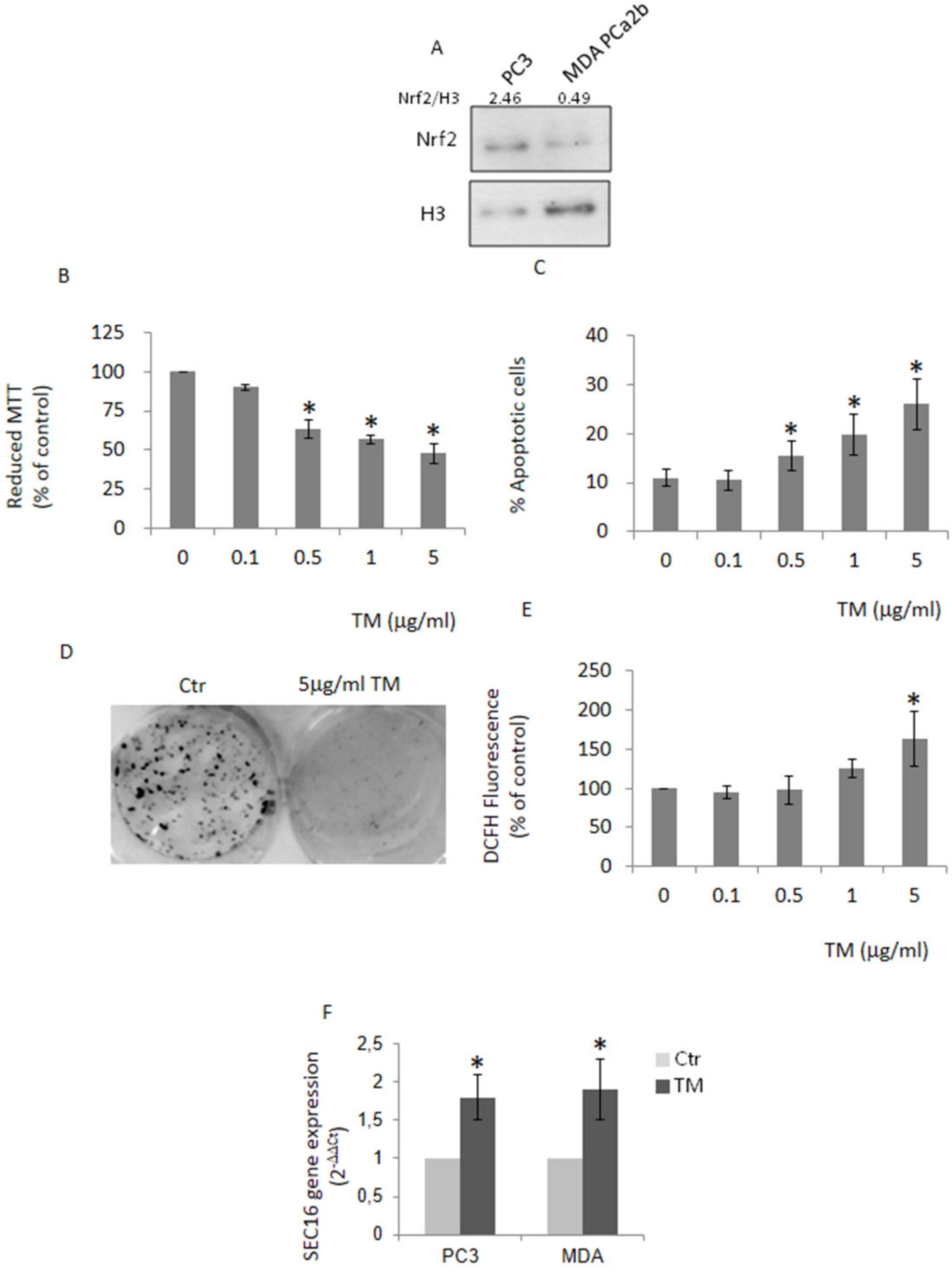

Supplementary Figure 1: PC3 and MDAPCa2b cells were grown under normal condition and nuclei used for detection of A) Nrf2 nuclear basal levels by western blotting, histone H3 was used as loading control. MDAPCa2b cells were treated with increasing concentrations (0-5µg/ml) of TM for 24 h. B) Cell viability as detected by MTT assay; C) percentage of apoptotic cells as detected by PI staining and FACS analysis; D) clonogenic assay, in the presence of 5µg/ml TM; E) ROS levels as detected by DCFH fluorescence. F) SEC16 expression in PC3 and MDAPCa2b cells, as determined by qPCR. Expression was normalised to GAPDH and reported as $2^{-\Delta\Delta Ct}$. Relative mRNA level of untreated cells was assumed to be 1. *$p < 0.05$ vs. control cells.

Supplementary Table 1: List of antibodies

Antibody (clone)	Dilution	Company
Bip/GRP78 (40)	1:2000	BD Transduction Laboratory
p-eIF2α (ser 51)	1:1000	Cell Signalling
p-IRE1α (Ser 724)	1:500	Thermo Scientific, Rockford, IL
IRE1α	1:1000	Cell Signalling
p-ERK1/2 (Thr202/Tyr 204)	1:1000	Cell Signalling
p-p38 (Thr 180/Tyr 182)	1:1000	Cell Signalling
p-SAPK/JNK (Thr 183/Tyr 185)	1:1000	Cell Signalling
p-Akt (Ser 473)	1:1000	Cell Signalling
p-GSK3β (Ser 9)	1:1000	Cell Signalling
Nrf2 (A10)	1:200	Santa Cruz Biotechnology
p-NF-κB p65 (Ser 536)	1:1000	Cell Signalling
Lamin B (H300)	1:200	Santa Cruz Biotechnology
GAPDH	1:500	Santa Cruz Biotechnology
Anti-rabbit IgG HRP	1:5000	Santa Cruz Biotechnology
Anti-goat IgG HRP	1:5000	Santa Cruz Biotechnology
Anti-mouse IgG HRP	1:5000	Santa Cruz Biotechnology

Supplementary Table 2: List of primers

Gene name	Gene symbol	Primer sequence (F: Forward; R: Reverse)
Hemeoxygenase-1	HO-1	F: TGTGGCAGCTGTCTCAAACCTCCA<br>R: TGAGGCTGAGCCAGGAACAGAGT
NADPH:quinine oxidoreductase	NQO1	F: GGGTATCTTTCCAGGCTTCC<br>R: TTTCTACATCTTTCCCTAAGTGGC
Glyceraldehyde 3-phosphate dehidrogenase	GAPDH	F: TGGTATCGTGGAAGGACTCATGAC<br>R: ATGCCAGTGAGCTTCCCGTTCAGC
Endoplasmic reticulum export factor	SEC16	F: CCCGTAGGAGGTGAAACAGA<br>R: CGATCTGCCTCAAATGGTTT
